# *In Vitro* Evaluation and Mechanism Analysis of the Fiber Shedding Property of Textile Pile Debridement Materials

**DOI:** 10.3390/ma9040302

**Published:** 2016-04-22

**Authors:** Yijun Fu, Qixue Xie, Jihong Lao, Lu Wang

**Affiliations:** Key Laboratory of Textile Science and Technology of Ministry of Education and College of Textiles, Donghua University, Shanghai 201620, China; fyjviolin@163.com (Y.F.); xie_qx0124@163.com (Q.X.); ljh@dhu.edu.cn (J.L.)

**Keywords:** textile pile debridement material, fiber shedding, single fiber pull-out test, structural design, back-coating, failure mode

## Abstract

Fiber shedding is a critical problem in biomedical textile debridement materials, which leads to infection and impairs wound healing. In this work, single fiber pull-out test was proposed as an *in vitro* evaluation for the fiber shedding property of a textile pile debridement material. Samples with different structural design (pile densities, numbers of ground yarns and coating times) were prepared and estimated under this testing method. Results show that single fiber pull-out test offers an appropriate *in vitro* evaluation for the fiber shedding property of textile pile debridement materials. Pull-out force for samples without back-coating exhibited a slight escalating trend with the supplement in pile density and number of ground yarn plies, while back-coating process significantly raised the single fiber pull-out force. For fiber shedding mechanism analysis, typical pull-out behavior and failure modes of the single fiber pull-out test were analyzed in detail. Three failure modes were found in this study, *i.e.*, fiber slippage, coating point rupture and fiber breakage. In summary, to obtain samples with desirable fiber shedding property, fabric structural design, preparation process and raw materials selection should be taken into full consideration.

## 1. Introduction

Skin wounds with various characteristics are a global issue and a major threat to the public health and economy [1,2,3]. Those that fail to heal within an anticipated time and do not proceed through a highly organized reparative process, which results in anatomic and functional integrity, are considered chronic [2,4]. Pressure, venous and diabetic foot ulcers are the three main categories of chronic wounds [5,6,7]. To solve these problems, the primary step in chronic wound treatment is to achieve effective debridement or wound bed preparation. Wound debridement is the medical removal of devitalized, damaged, infected or contaminated tissue from the wound bed to improve the healing potential for the remaining healthy tissue [8,9]. It is the main tool for maintaining a healthy wound bed in most chronic wounds and a recognized component of good wound care as it reduces the bioburden of the wound and improves the life quality of patients [10,11]. The TIME concept (Tissue, Inflammation (or Infection), Moisture, Edge) proposed by European Wound Management Association (EWMA) in 2003 has been widely accepted as a practical guide for the debridement of chronic wounds [12,13].

Inflammation, a natural physiological reaction in the circumstance of wounds, is a normal stage in the wound healing process. However, intensive or inappropriate inflammation always results in infection, which complicates the wound, impedes wound healing, and increases patient discomfort and/or anxiety [14,15]. To control the inflammation or infection, a totally removal of the necrotic tissues is requisite in the procedure of debridement. Meanwhile, new pollutant or foreign body should not be introduced into the wound bed. Among various debridement methods, medical cotton and absorbent gauze are the most commonly seen biomedical textile materials, which can be used as either a swab or patch to clean wounds [16,17]. They are preferred due to the low cost and easy operating [18,19]. However, fiber shedding phenomenon was reported when a gauze was attached or detached during the wet-to-dry debridement [20,21]. The shed fibers remained in the wound bed not only contaminate the microenvironment but also increase the susceptibility of infection [20]. Thus, fiber shedding property is an indispensable aspect of any materials designed for wound debridement application.

A monofilament fiber pad has been recently introduced as a new solution to offer quick, effective and non-traumatic wound debridement for patients suffering from chronic wound [22,23]. It was reported in our previous study that this material exhibited sufficient mechanical properties, superior liquid absorption and satisfactory biocompatibility [24,25]. However, since this material is a kind of knitted pile fabric consists of abundant single fibers in the face side which has a direct contact with the wound site. A close look into the fiber shedding property of this debridement material is of great necessity.

Several testing methods are available to evaluate the fiber shedding propensity of textile fabrics. The modified Gelbo Flex method is a normative method used to assess the lint and particles generated from medical textile products in dry state, such as surgical drapes, gowns and protective clothing [26,27]. The fabric sample is subjected to a combined twisting and compression action in a test chamber. Number of particles released from the testing fabric is counted and classified using a particle counter within the size range from 0.3 to 25 μm. Another test method for evaluation of the wet linting in nonwoven dressings is introduced in the EN 1644-2 [28]. The test sample is firstly shook in water at the frequency of 300–350 Hz for 10 min. Then, fibers shed from the dressing are collected using a gridded filter. Number of shed fibers is counted and taken as the result. Other testing methods, such as abrading and tape methods, are also reported to estimate the fiber shedding propensity of the apparel or garment textiles [29,30,31]. Though the detailed operation modes of these testing methods are diverse from each other, they all aim to simulate the type of motions that may cause lint or particles during application. However, results of all these tests are only presented as the amount (number and/or mass) of the loosing fibers. Thus, profound analysis on fiber shedding mechanisms is hardly to be achieved through these methods.

Therefore, the aim of this study was first to propose an *in vitro* testing method, which can be employed to evaluate the fiber shedding property of the textile pile debridement material. Furthermore, influence of structural design (pile density, number of ground yarns and back-coating time) on the fiber shedding property of the debridement material was explored. In addition, the typical load–displacement curve and failure modes of the single fiber pull-out test were investigated to better understand the fiber shedding mechanisms of this textile pile debridement material.

## 2. Results and Discussion

### 2.1. Microstructure

**Figure 1:**
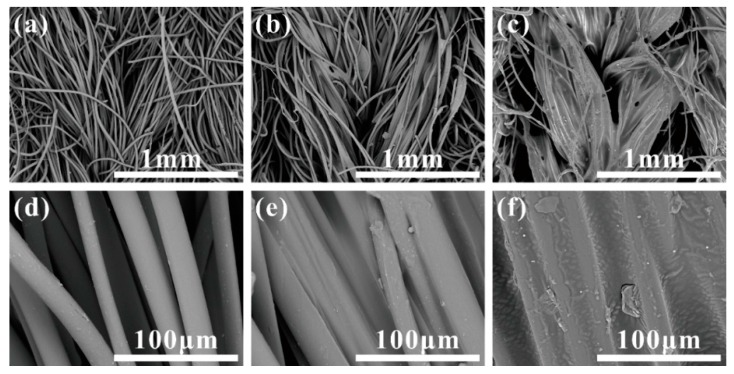
SEM images of the backside for different textile pile materials: (**a**,**d**) H3-0; (**b**,**e**) H3-1; and (**c**,**f**) H3-2.

The morphological structure of the backside of samples H3-0, H3-1 and H3-2 are presented in Figure 1. The ground yarns were regularly arranged and formed the single jersey stitch on both samples before and after back-coating (Figure 1a–c). Fibers with smooth surface were distributed independently under high magnification for sample H3-0 (Figure 1d), whereas the filling adhesive partially covered the fibers and the gaps among each other for H3-1, leaving some fibers still visible (Figure 1e). As for sample H3-2, a continuous membrane structure was clearly observed, which totally covered the adjacent fibers (Figure 1f). This was caused by the difference in back-coating repetitions.

### 2.2. Surface Chemistry Analysis

**Figure 2:**
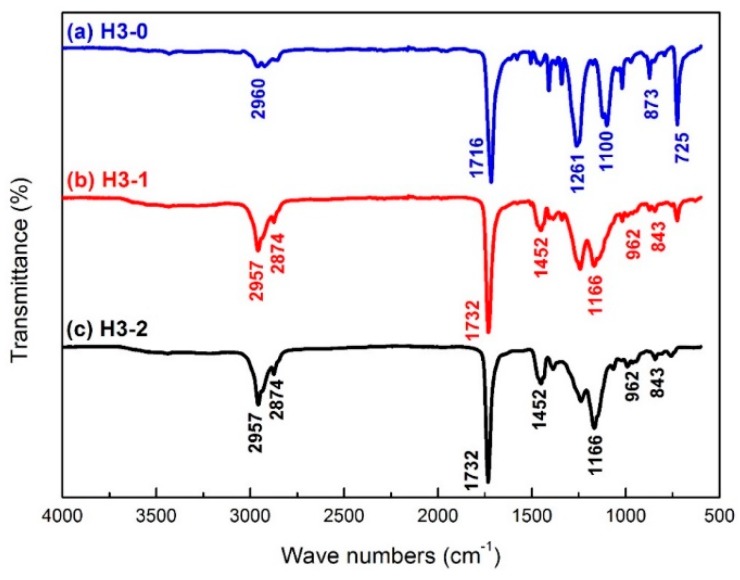
FTIR spectra of the backside for different textile pile materials: (**a**) H3-0; (**b**) H3-1; and (**c**) H3-2.

Chemical changes on the backside of samples before and after back-coating were detected by FTIR and the spectra of samples H3-0, H3-1 and H3-2 are shown as curve a, b and c in Figure 2, respectively.

In the FTIR spectrum of sample H3-0 (Figure 2a), the characteristic absorption peak at 1716 cm^−1^ can be assigned to the strong symmetric stretching of carbonyl groups C=O [32]. The absorbance around 1261 and 1100 cm^−1^ are attributed to the stretching vibration of C–O group [33]. The bands appearing at the frequency of 2960, 873 and 725 cm^−1^ are the C–H stretching, C–C out of plane bending and C–H bending vibrations of the benzene rings in polyester, respectively [34].

After back-coating treatment, the peaks around 2957 cm^−1^ were noticeably intensified and a new peak can be seen at 2874 cm^−1^ in both samples H3-1 and H3-2 (Figure 2b,c). These are the asymmetric and symmetric vibration of C–H bonds in methylene group CH_2_ [35,36]. Compared with the spectrum of sample H3-0 (Figure 2a), there were no evident absorption peaks around 1100, 873 and 725 cm^−1^ in the spectra of samples with back-coating (H3-1 and H3-2). In addition, both H3-1 and H3-2 exhibit new characteristic absorption peaks: stretching of the carbonyl group C=O around 1732 cm^−1^ [35,36], distortion vibration of CH_2_ at 1452 cm^−1^ [37], and the stretching vibration of C–O–C, C–C and C=O in the acrylic group at 1166, 962 and 843 cm^−1^, respectively [38]. All these characteristic absorption peaks indicate that the polyacrylate latex was successfully coated on the backside of samples H3-1 and H3-2.

When comparing the FTIR spectra of H3-1 and H3-2, it is interesting to note that the absorption peaks at 2957, 1452 and 1166 cm^−1^ for H3-2 were more intensive than those of H3-1. This implies that H3-2 had a relatively high amount of polyacrylate latex coated onto its back surface after two back-coating treatments compared to H3-1.

### 2.3. Fabric and Pile Weight per Unit Area

**Figure 3:**
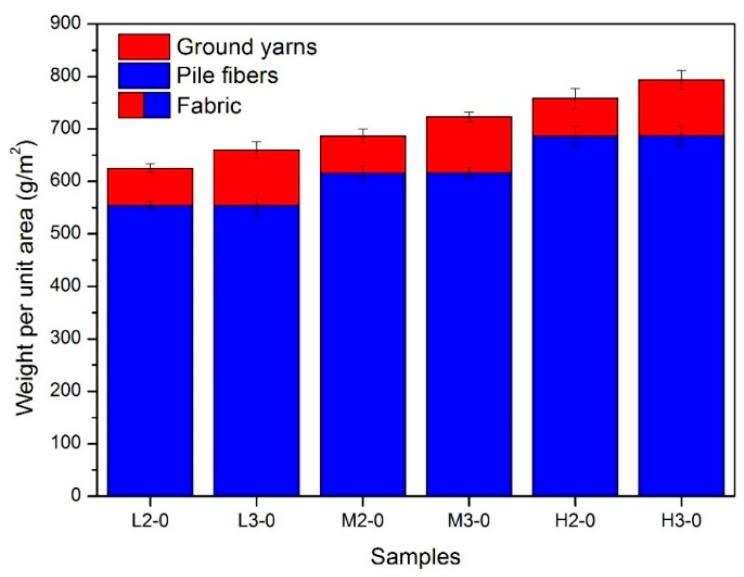
Results of fabric and pile weight per unit area for different samples.

The weight per unit area of ground yarns (red), pile fibers (blue) and total fabric (red plus blue) are given in Figure 3, from which we can see that the fabric weight consisted of two parts, *i.e.*, pile and ground yarns weight. Moreover, pile weight accounts for almost 90% of the total fabric weight. The fabric weight per unit area of six samples shown a slight uptrend from 624.79 g/m^2^ for sample L2-0 to 793.62 g/m^2^ for sample H3-0. When controlling the processing parameters, similar pile weights per unit area were obtained for samples designed with identical pile density. More specifically, the pile weight per unit area of samples with low, middle and high pile density (samples L2-0, L3-0, samples M2-0, M3-0 and samples H2-0, H3-0) were about 554, 616 and 687 g/m^2^ individually. This implies that pile weight per unit area can be precisely controlled by adjusting the preparation technological parameters, and it is a more appropriate index to reflect the designed pile density, rather than the total fabric weight per unit area.

### 2.4. Characterizations of the Ground Fabric

#### 2.4.1. Morphology Observation

**Figure 4:**
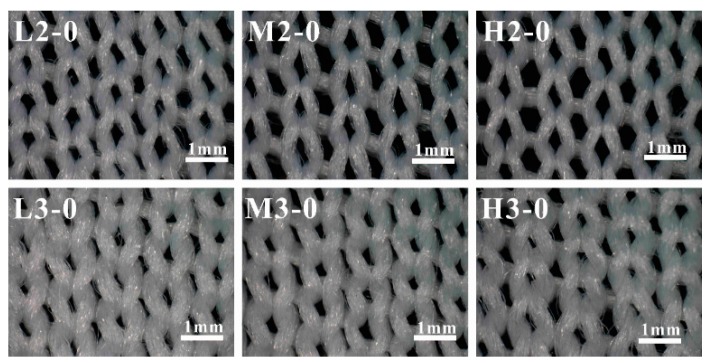
Light microscope photos of the ground fabric for different samples.

**Figure 5:**
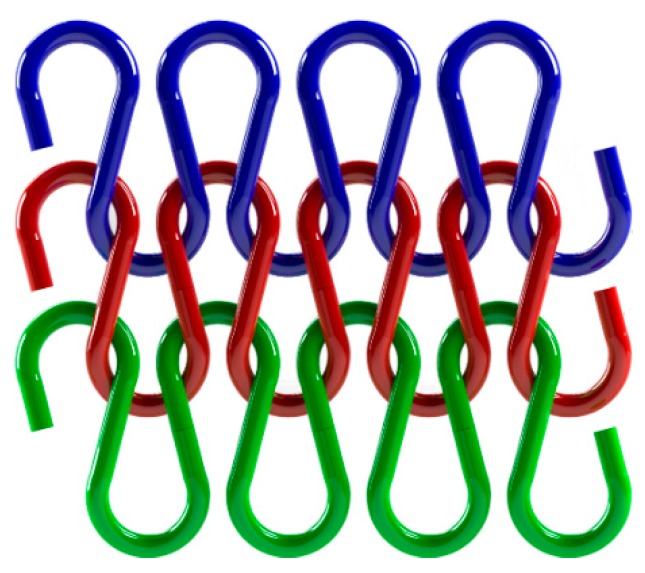
3D diagram of the ground fabric.

Light microscope images of the ground fabric of six pile materials are presented in Figure 4. The ground fabric structure of all the samples is a single jersey stitch that consisted of continuous loops in weft direction. To better illustrate the ground fabric structure, a 3D diagram was drawn in Figure 5 where the well-constructed stitches were clearly observed. Unlike the regular arranged stitches in the 3D diagram, the pores formed by the ground yarns were in various shapes and sizes as shown in Figure 4. Besides, the knitted stitches were more compact in their real status, the loop head was covered by the two side limbs of previously formed loop. Generally, samples with three plies of ground yarns (L3-0, M3-0 and H3-0) are much tighter compared to samples with two plies of ground yarns (L2-0, M2-0 and H2-0).

#### 2.4.2. Surface Yarn Coverage

**Figure 6:**
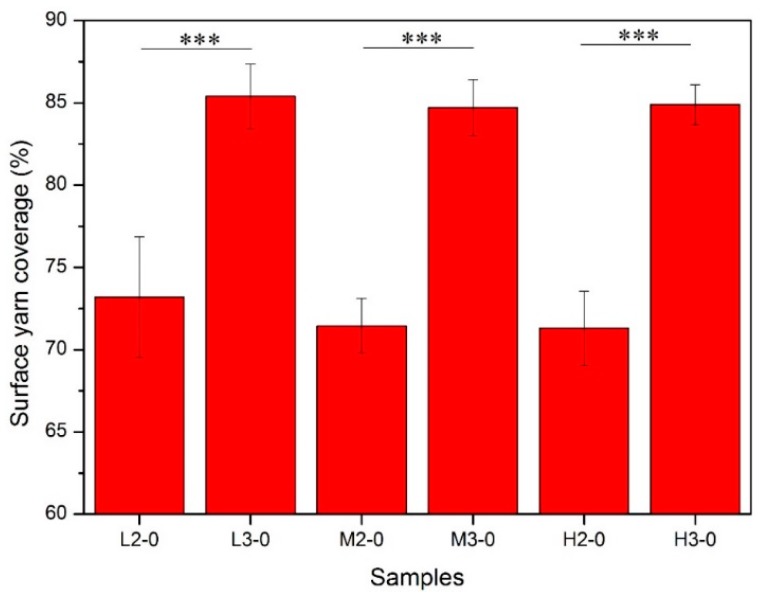
Surface yarn coverage of the ground fabric for different samples. (Significant differences were marked by *** for *p* ≤ 0.001).

Corresponding to the qualitative observation results of ground fabric, difference in tightness was observed among the six samples. In order to quantitatively describe this characteristic of the ground fabric, surface yarn coverage was calculated and compared among six samples. As presented in Figure 6, it is evident that the surface yarn coverage for samples with three plies of ground yarns (L3-0, M3-0 and H3-0) are about 85%, while samples with two plies of ground yarns (L2-0, M2-0 and H2-0) have a value around 71%. One-way analysis of variance (ANOVA) gives the same results: the surface yarn coverage of samples in these two groups are significantly different at the 0.001 level, while no statistical differences at 0.05 confidence level were observed within each group. This implies that the number of ground yarns was the controlling structural factor that influenced surface yarn coverage of the ground fabric.

#### 2.4.3. Distribution of the Stitch Size

**Figure 7:**
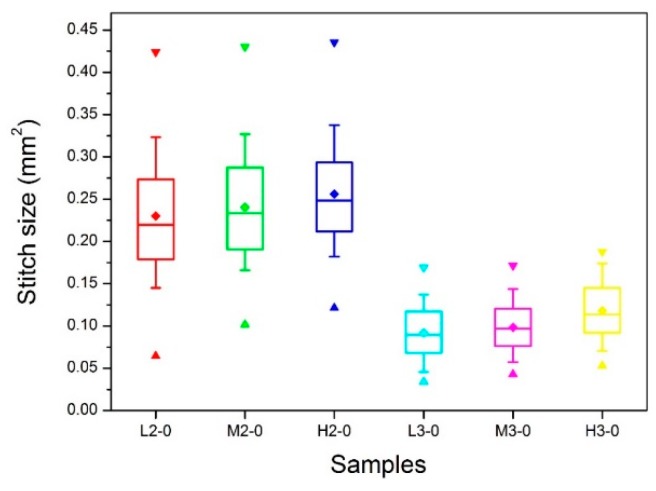
Stitch size distribution of the ground fabric for different samples.

As mentioned above, the knitted stitches for six samples were in various shapes and sizes (Figure 4). To quantitatively analyze stitch size, a total of 100 stitches were measured on each sample and the distribution of stitch size was assessed via box chart plotted in Figure 7. The bottom, internal, and top bands of the box represent the first quartiles (25%), median value (50%), and third quartiles (75%), respectively [39,40]. The lower and upper ends of the whisker are the 10th and 90th percentiles of the distribution, respectively. Furthermore, the minimum, maximum and average value of the data are shown as regular triangle, inverted triangle and rhombus, respectively. To acquire an insight into the stitch size distribution, the most relevant statistical parameters are summarized in Table 1, which includes the average (Ave.), minimum (Min.) and maximum (Max.) values, as well as the peak values range (PVR) [41].

As can be seen from Figure 7 and Table 1, there is a wide distribution of stitch size for samples with two plies of ground yarns varied from 0.31 (H2-0) to 0.36 mm^2^ (L2-0), whereas, samples with three plies of ground yarns exhibited a relatively narrow distribution about 0.13 mm^2^. Moreover, the median value for samples with two plies of ground yarns can reach 0.26 mm^2^ in H2-0, which is much bigger than that of samples with three plies of ground yarns (around 0.10 mm^2^). This suggests that the number of ground yarns are dominant factor influencing the stitch size. However, it is also interesting to notice that there is a positive correlation between stich size and pile density for samples with the same number of ground yarns. That is to say, among samples with two plies of ground yarns, L2-0 has the lowest average value, H2-0 has the highest vale, and M2-0 has value in between. Same trend can be found in L3-0, M3-0 and H3-0.

### 2.5. Single Fiber Pull-Out Test

**Figure 8:**
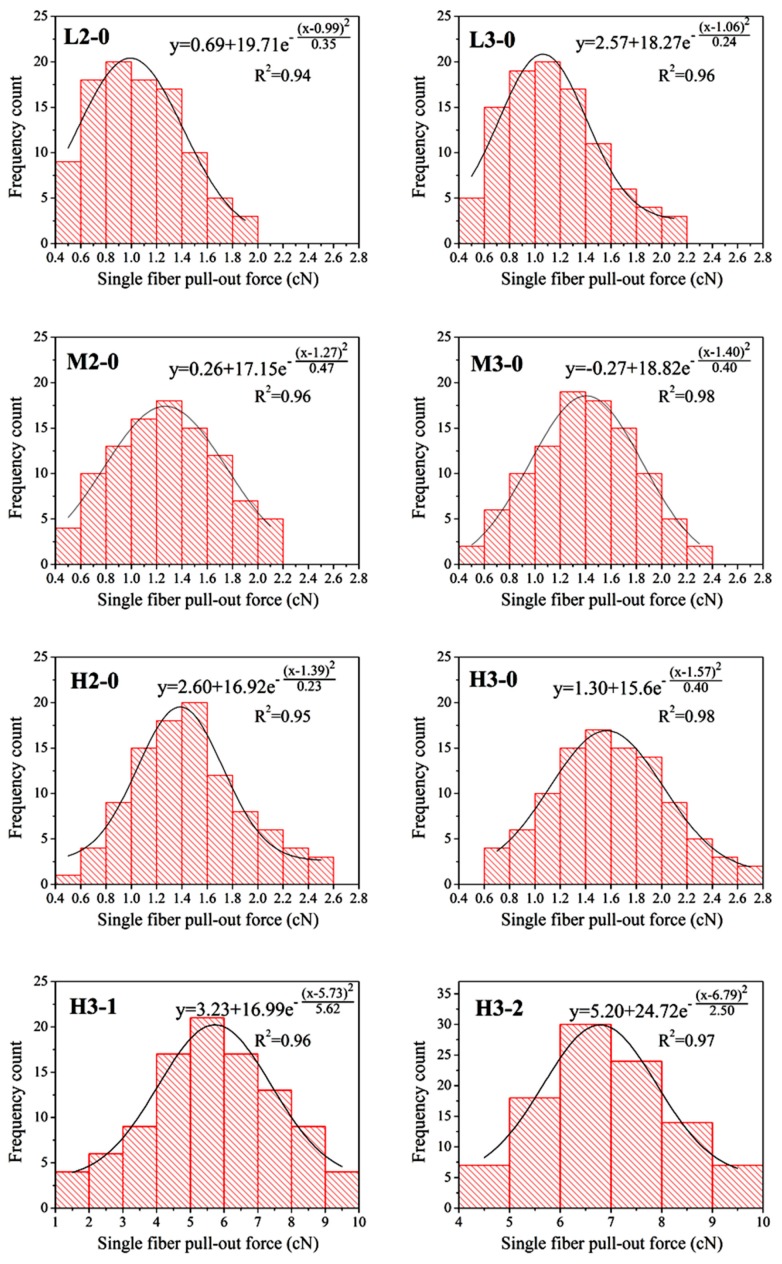
Frequency distributions (histograms) and GaussAmp simulation (black curve) of single fiber pull-out force for different samples.

The maximum pull-out load value was defined as single fiber pull-out force to describe the pull-out behavior of the testing samples. A high pull-out force indicates a good consolidation between the ground fabric and pile fibers, as well as a low fiber shedding probability during application. The initial data of single fiber pull-out force for different textile debridement materials were processed by frequency histograms displayed in Figure 8. GaussAmp function (Equation (1)) was selected to fit the data by the Origin software shown as black curve in Figure 8.

(1) $$y=y_{0}+Ae^{-\frac{{\left(x-x_{c}\right)}^{2}}{2w^{2}}}$$

where *y*_0_ is the offset, *A* is the amplitude, *x*_c_ is the axial coordinate of the peak, and *w* is related to the full width at half maximum (FWHM) [42,43]. The fitted parameter values and correlation coefficients of each figure are listed in Table 2.

It can be clearly seen that the GaussAmp distribution model was apposite to describe all the results with correlation coefficients above 0.94. For each kind of sample, there is a certain distribution of single fiber pull-out force. For example, single fiber pull-out force of sample L2-0 laid within the 0.4–2.0 cN range. This can be associated with the wide distribution of stitch size inside each sample (Figure 7), which made for difference in frictional resistance between pile fiber and ground yarns. Furthermore, the pull-out force of a single fiber withdrawn from the debridement material may be affected by other factors, such as fiber orientation, fiber crimp and fiber position in the pile tuft [44].

#### 2.5.1. Influence of Structures on the Single Fiber Pull-Out Force

##### Pile Density

The curve center (*x_c_*) of samples L2-0, M2-0 and H2-0 moved towards the right along the *x*-axis from 0.99 to 1.27 then to 1.39 cN (Table 2). This can be explained by the difference in pile density, *i.e.*, the pile weight per unit area (Figure 3). On the basis that all the six samples having the same stitch density, the higher the pile weight per unit area, the more the amount of pile fibers per stitch. Consequently, the inter-fiber squeezing action and cohesive force among pile fibers in sample H2-0 was stronger compared to that of M2-0 and L2-0, which resulting in a higher single fiber pull-out force for H2-0. Drift in the same direction can also be found among samples with three plies of ground yarns before back-coating. The peak value of single fiber pull-out force for M3-0 and H3-0 were 32.08% and 48.11% higher than the peak value for L3-0, respectively. It can be summarized that higher pile weight per unit area leads to increased single fiber pull-out force and decreased fiber shedding propensity. Therefore, high pile weight per unit area is preferred when design the fabric structure for textile pile materials for debridement application.

##### Number of Ground Yarns

On the other hand, ground yarn plies also played an evident role in impacting the single fiber pull-out force of textile pile debridement materials. Compared to samples with two plies of ground yarns, the single fiber pull-out force of samples L3-0, M3-0 and H3-0 increased 7.07%, 10.24% and 12.95%, respectively. This is corresponding to the results of surface yarn coverage (Figure 6) and stitch size (Figure 7). The change in the number of ground yarns from two to three lead to apparent raise in surface yarn coverage and reduce in stitch size. In other words, the ground fabric became tighter with the supplement of ground yarn plies. Thus, the squeezing pressure on pile fibers provided by ground fabric was enhanced, which resulted in a higher single fiber pull-out force. For this reason, ground yarn plies should be designed at a relative high level in order to obtain textile pile debridement materials with suitable single fiber pull-out force and desirable fiber shedding property.

##### Back-Coating

Considering the influence of back-coating treatment on single fiber pull-out force, the *x_c_* values of H3-1 (5.73 cN) and H3-2 (6.79 cN) are more than three times higher than that of H3-0 (1.57 cN), which indicates that back-coating treatment was an effective approach to improve the single fiber pull-out force. As shown in Figure 1e, a partial coverage of the fiber surface was noticeable on sample H3-1, which only experienced one time back-coating treatment, while a nearly complete filling of the fiber interspaces was achieved on sample H3-2 after two back-coating treatments (Figure 1f). The procedure of back-coating not only changed the surface structure, but also offered a chemical combination between the pile fibers and the ground yarns. It is the back-coating process that changed the binding mode between pile fibers and ground yarns from mechanical fixation into chemical combination. Therefore, back-coating is an indispensable process of the textile pile debridement preparation. Samples after back-coating treatment shown a substantial enhancement in single fiber pull-out force and fiber shedding property.

#### 2.5.2. Mechanism Analysis

##### Samples without Back-Coating

**Figure 9:**
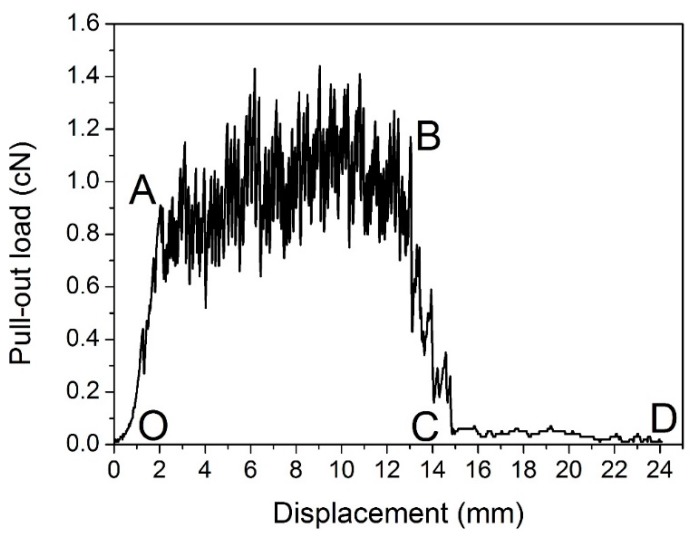
Load–displacement curve obtained from samples without back-coating (H3-0).

**Figure 10:**
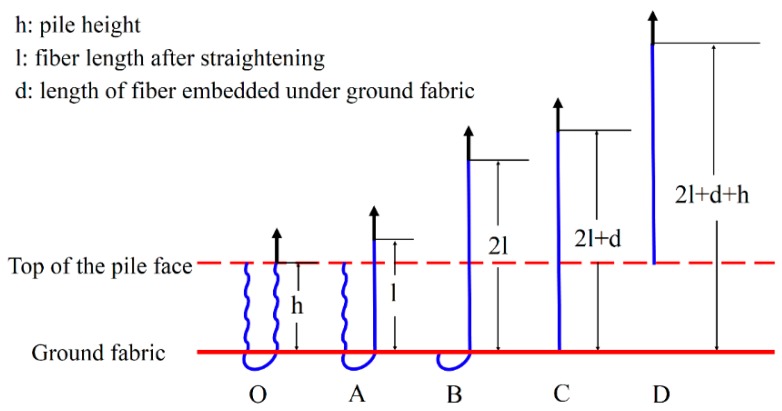
Schematic diagram of the single fiber pull-out process.

The load *vs.* displacement curve plotted in Figure 9 corresponds to the generally observed single fiber pull-out behavior of samples without back-coating (H3-0). In this representative curve, some relevant points were marked with letters to better illustrate the pull-out process elaborated in Figure 10.

In Figure 9, a dramatic rise in pull-out load was observed from the origin point O to point A, which can be attributed to the fiber straightening in the initial response [45]. The serrated shape occurred during AB section was due to the friction and sliding between the extracted fiber and its surrounding fibers in the pile tufts under the knitted stitches of the ground fabric [46]. Afterwards, the profile experienced a fluctuating decline from point B to C as the length of anchored fiber under the knitted fabric and the number of fiber-to-fiber interactions decreased [47]. In the last CD stage, the pull-out load was maintained at a relative low level for the reason that there was only loose contact between the extracted fiber and its adjacent pile fibers in vertical direction. The displacement between point C and D was about 10 mm, which is approximate to the pile height of the samples. All these characteristics conclude that the bond strength between ground fabric and pile fibers is quite lower than the tensile strength of the pile fiber itself in samples without back-coating. This can be explained by the binding mode between pile fibers and ground yarns. Since there was no back-coating on the backside, the consolidation of pile fibers was only provided by the mechanical combination from ground yarns. Thus, an intact pile fiber was withdrawn from the fabric at a rather low level of external force [47].

Figure 10 gives a clear illustration of the entire process of the single fiber pull-out test. The single fiber pull-out behavior on samples without back-coating was composed of three different mechanisms: fiber straightening (OA), fiber friction and sliding under the ground fabric (AB and BC), and fiber frictional slip among vertical pile fibers (CD) [48].

##### Samples with Back-Coating

Three distinct responses, namely fiber slippage, coating point rupture and fiber breakage, were found during the single fiber pull-out test on samples with back-coating. Representative load–displacement curve, fracture morphology and schematic diagram of the pull-out process of each failure mode are presented in Table 3.

The load–displacement curve of fiber slippage mode was similar to that of H3-0 (Figure 9). This suggests a pretty weak bonding point where the pull-out load was sufficiently stronger than the consolidation between pile fiber and its surrounding coating material [47,49]. SEM observations revealed that the slipping fiber was intact but some residual coating was found along the fiber surface. The coating remaining on the fiber increased the friction resistance during the pull-out procedure. Thus, the AB section of fiber slippage mode was fluctuated at a higher level (1.0–2.0 cN) than that of sample H3-0 (0.6–1.4 cN).

Unlike the fiber slippage mode that exhibited a profile characterized with frequent fluctuations, the load–displacement curve of coating point rupture mode was relative smooth. It can be observed that the pull-out curve was comprised of two approximately linear parts (OA and AB) before the crest value was achieved. Then, a marked drop in load occurred immediately after the peak point B (around 4.8 cN), indicating a simultaneous fracture at the binding site of the pile fiber and its circumambient coating material (disconnection of the green line in the pull-out process C) [49,50]. Besides, a broken fiber end wrapped with a thick layer of coating material was observed from the SEM image of coating point rupture mode, which confirms the failure mechanism described above.

The load–displacement relation for fiber breakage mode displayed an almost linear increase (OA) at the beginning. Then, the load suddenly dropped to point B, which suggests partial damage occurred in the middle of the fiber length (blue dash line in the pull-out process B) [51,52]. Afterward, the remaining part of the fiber continued to take the pull-out load and the curve raised from point B to C (dash-dot line in the pull-out process C), then reach to the maximum value at point D (above 8 cN) followed by a straight descending (complete disconnection of the dot line in the pull-out process E). The SEM micrograph of fiber breakage mode shown a fiber cracked into two parts, which corresponds to the two fallings phases (AB and DE) occurred in the load–displacement curve. All these characteristics indicate an ideal point where the chemical consolidation force between pile fiber and its surrounding coating materials was stronger than the pile fiber strength. At this circumstance, the single fiber pull-out force was mainly relied on the tensile strength of polyester pile fiber used as raw materials. In another words, the properties of the pile fiber, such as fiber critical length, also played an important role in affecting the mechanical properties of the textile pile debridement materials [53].

According to the description above, the 100 results of single fiber pull-out test for each sample can be classified into three failure modes. In addition, the pull-out force required for different modes show an uptrend in the order of fiber slippage, coating point rupture and fiber breakage. The percentages of each failure mode for samples with different back-coating repetitions are listed in Table 4. It shows that all the 100 fibers pulled from H3-0 belong to the category of fiber slippage, while the major failure mode for samples H3-1 and H3-2 was fiber breakage. Results illustrated in Table 4 denote that the back-coating process is effective in improving the single fiber pull-out force for textile pile debridement materials. That is to say, the fiber shedding phenomenon can be well controlled via back-coating treatment, which considerably reduces the probability of wound contamination and infection. However, fiber breakage still occurred for samples with two back-coatings, which was due to the limitation of raw material strength. Therefore, in order to obtain samples with desirable fiber shedding property for wound debridement application, fabric structural design, preparation process and raw materials selection should be taken into full consideration.

## 3. Materials and Methods

### 3.1. Materials

Polyester staple fiber and multifilament yarn (Jiangsu Chemical Fiber Co., Ltd., Suqian, China) were selected as the raw materials to fabricate the textile pile fabric. Table 5 lists some general information of these raw materials. A biocompatible polyacrylate latex was also coated onto the backside of the pile material during the procedure of post treatment.

### 3.2. Design and Fabrication of the Textile Pile Debridement Material

**Figure 11:**
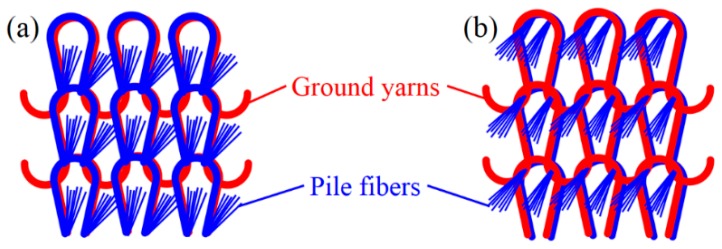
Single jersey stitch structure of the pile fabric: (**a**) backside view; and (**b**) pile side view.

Eight prototype samples of the textile pile materials were designed in this study. The structural parameters for each sample, including pile density, ground yarn plies and back-coating time, are listed in Table 6, together with the pile height and stitch density. All the samples were knitted with the same single jersey stitch structure, as shown in Figure 11. From the backside view (Figure 11a), the pile fibers (blue) were physically anchored by the ground fabric (red), while, seen from the face side (Figure 11b), typical knitting loops formed by the ground yarns were clearly observed and two ends of the pile tufts vertically extended out of the ground fabric to form the U shape pile face.

All the samples were firstly knitted on a special circular machine (SK18, Mayer Industries Inc., Tailfingen, Germany) under ambient conditions of 20 °C and 60% RH. Then, the pile face went through a cutting machine and the pile fibers were trimmed to the height of 10 mm, as designed in Table 6. After cutting, the backside of the samples were coated with biocompatible polyacrylate latex and dried in an oven.

### 3.3. Microstructure

Scanning electron microscopy (SEM) was employed to investigate the surface characteristics of the textile pile materials. The micrographs of the backside of samples before and after back-coating were obtained using a TM-3000 SEM (Hitachi, Tokyo, Japan) with an accelerating voltage of 15 kV.

### 3.4. Surface Chemistry Analysis

Fourier transform infrared spectrometer (FTIR) analysis was performed on a Nicolet 6700 FTIR (Thermo Fisher Scientific, Waltham, MA, USA) to study the surface chemistry of the backside of samples before and after back-coating in the range from 4000 to 500 cm^−1^. The spectra were analyzed using proprietary software (Omnic V 7.3, Thermo Fisher Scientific, Waltham, MA, USA, 2006).

### 3.5. Fabric and Pile Weight per Unit Area

Five specimens measuring 10 cm × 10 cm were conducted under standard condition for 24 h before weighting on an analytical balance [54]. Then the pile fibers were carefully detached from the ground yarns, collected and weighted. Special attention was paid to avoid fiber losing during this procedure. Both fabric and pile weight per unit area were calculated as Equations (2) and (3) [55], respectively.

(2) $$W_{f}=\frac{W_{1}}{A}$$

(3) $$W_{p}=\frac{W_{2}}{A}$$

where *W_f_* and *W_p_* are the fabric and pile weight per unit area in g/m^2^, *W*_1_ and *W*_2_ are the weight of the total fabric and collected pile fibers (g) in a testing area of *A*, which is 0.01 m^2^ in this study.

### 3.6. Characterizations of the Ground Fabric

#### 3.6.1. Morphology Observation

Samples with a size of 10 cm × 10 cm were conducted under standard condition for one day [54]. Then, the pile fibers in the central of the samples were carefully extracted out of the loops formed by ground yarns to give an exposed area of ground fabric measuring 2 cm × 2 cm. Afterwards, the exposed area was taken for morphology observation under a PXS8-T stereoscopic microscope (Olympus, Takachiho, Japan).

#### 3.6.2. Surface Yarn Coverage

**Figure 12:**
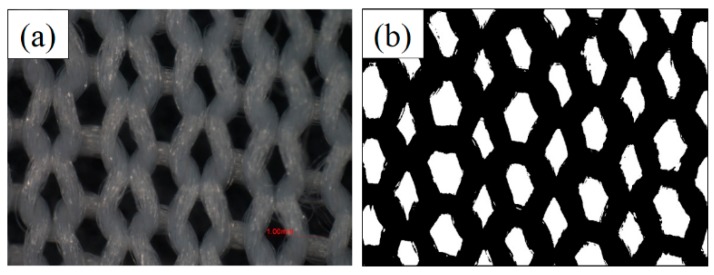
Original photo (**a**) and binary image (**b**) used to calculate the surface yarn coverage of the ground fabric.

The photomicrographs of the ground fabric were analyzed by Image J software (Version 1.49q, National Institutes of Health, Bethesda, MD, USA, 2015) to obtain the surface yarn coverage. The original light microphotograph (Figure 12a) was firstly transferred into binary image, as shown in Figure 12b. Then, the percent coverage of the ground yarn was calculated by dividing the pixelated value of the black area by that of the total area.

#### 3.6.3. Distribution of the Stitch Size

**Figure 13:**
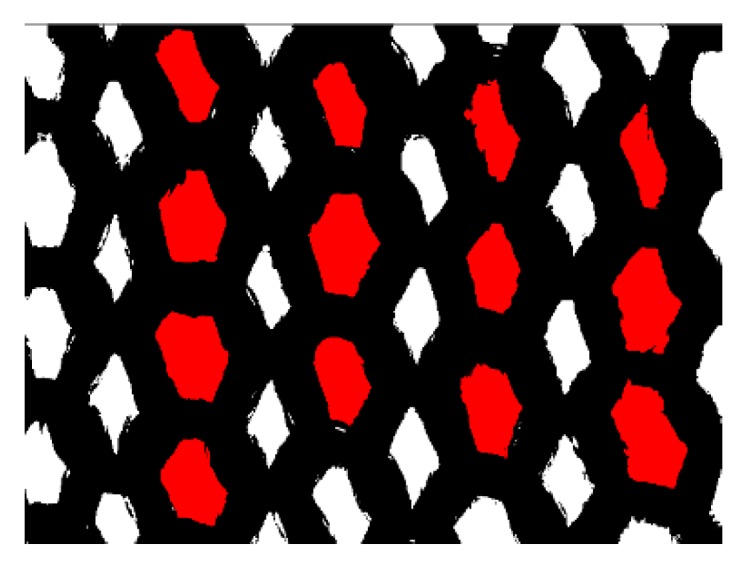
Measurement of the stitch size of the ground fabric.

Image Pro Plus software was used to measure the stitch size of the ground fabric. As shown in Figure 13, only the intact stitches that hold pile fibers were marked and measured (red parts). A total of 100 stitches were collected on each sample and the distribution of the stitch size was investigated.

### 3.7. Single Fiber Pull-Out Test

**Figure 14:**
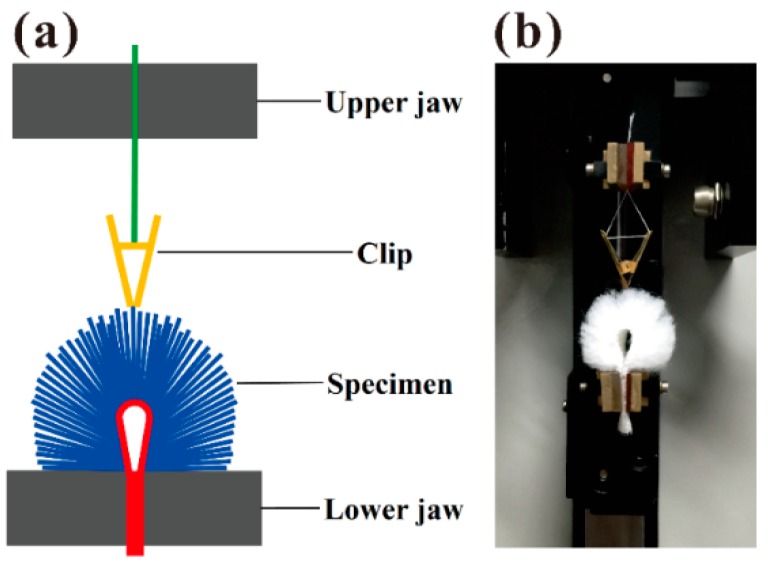
Schematic diagram (**a**) and photograph (**b**) of the single fiber pull-out testing setup.

Single fiber pull-out test is a well-recognized test for fiber composite [56]. In this study, an XQ-2 single fiber tensile tester (Shanghai Lipu Applied Science and Technology Research Institute, Shanghai, China) was employed to evaluate the single fiber pull-out behavior of the textile pile debridement materials at the stretching speed of 20 mm/min. The schematic diagram and photograph of the experimental system are illustrated in Figure 14. Testing specimen with a length of 4 cm and a width of 2 cm was folded at its length direction with pile fibers outward. The two ends of the specimen were snipped free of fibers, warped with scotch tape and held in the lower jaw. A clip was employed to clamp a single pile fiber stood upright from the ground fabric of the pile material. The other side of the clip was fixed with yarn, which was gripped by the upper jaw. A total of 100 pile fibers were extracted from each pile debridement material on 10 specimens.

### 3.8. Statistical Analysis

The results were analyzed statistically using Origin 8.5 software (Origin Lab Inc., Northampton, MA, USA). Statistical differences were determined by a one-way analysis of variance (ANOVA) and the means were considered significantly different at *p* ≤ 0.05 (*), *p* ≤ 0.01 (**) and *p* ≤ 0.001 (***).

## 4. Conclusions

In this research, single fiber pull-out test was proposed as an *in vitro* method to evaluate the fiber shedding property of the textile pile debridement materials. The influence of structures on the single fiber pull-out force was studied. By increasing pile density and number of ground yarns, a slight ascending trend in single fiber pull-out force was achieved. Moreover, back-coating treatment prominently enhanced the pull-out force of single fiber in pile materials. That is to say, back-coating is an effective means to improve the fiber shedding property of textile pile debridement materials. These results imply that textile pile debridement materials with back-coating have a relatively low probability for fiber shedding during wound debridement application. Thus, in order to avoid any unexpected infection caused by the shed fibers, back-coating treatment is highly recommended for the preparation of textile pile materials. To better understand the mechanisms of the fiber shedding phenomenon in textile pile debridement materials, a close look into the single fiber pull-out behavior and modes of failure were performed. Typical load–displacement curve of samples without back-coating were characterized with consecutive fluctuating, which indicates a totally extracted pile fiber. In contrast, three modes of failure were observed on samples with back-coating, namely fiber slippage, coating point rupture and fiber breakage. All findings in this study not only provide a feasible *in vitro* approach for fiber shedding evaluation, but also offer a guideline for designing and manufacturing of the textile pile materials that meet the basic clinical requirements for wound debridement application.

## Figures and Tables

**Table 1:** Summary of relevant parameters in the distribution of stitch size.

Parameters	L2-0	M2-0	H2-0	L3-0	M3-0	H3-0
Ave. (mm^2^)	0.23	0.24	0.26	0.09	0.10	0.12
Min. (mm^2^)	0.06	0.10	0.12	0.03	0.04	0.05
Max. (mm^2^)	0.42	0.43	0.44	0.17	0.17	0.19
PVR (mm^2^)	0.36	0.33	0.31	0.14	0.13	0.13

**Table 2:** Parameter values of fitted distribution of single fiber pull-out force for different samples.

Parameters	L2-0	M2-0	H2-0	L3-0	M3-0	H3-0	H3-1	H3-2
*y*_0_	0.69	0.26	2.60	2.57	−0.27	1.30	3.23	5.20
*A*	19.71	17.15	16.92	18.27	18.82	15.60	16.99	24.72
*x_c_*	0.99	1.27	1.39	1.06	1.40	1.57	5.73	6.79
*w*	0.42	0.48	0.34	0.34	0.45	0.45	1.68	1.12
*R*^2^	0.94	0.96	0.95	0.96	0.98	0.98	0.96	0.97

**Table 3:** Three failure modes of single fiber pull-out test on samples with back-coating.

Items	Fiber Slippage	Coating Point Rupture	Fiber Breakage
Representative load–displacement curve	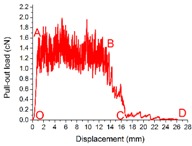	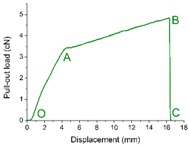	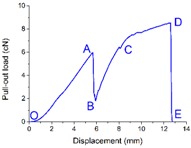
Fracture morphology	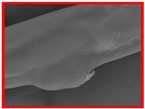	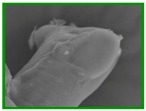	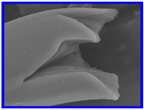
Schematic diagram of the single fiber pull-out process	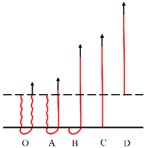	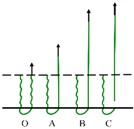	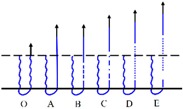

**Table 4:** Percentage of each failure mode for samples with different back-coating repetitions.

Sample No.	Fiber Slippage (%)	Coating Point Rupture (%)	Fiber Breakage (%)
H3-0	100	0	0
H3-1	10	26	64
H3-2	0	7	93

**Table 5:** Raw materials used to fabricate textile pile fabric.

Raw Materials	Linear Density	Number of Filaments per Yarn	Maximum Tensile Force (cN)	Elongation (%)
Staple fiber	3D ^1^	-	8.17 ± 0.59	48.05 ± 9.34
Multifilament yarn	150D ^1^	36	533.01 ± 21.13	28.29 ± 0.70

^1^ D = denier (mass in g of 9000 m length).

**Table 6:** Fabric structures of eight prototype samples.

Sample No.	Pile Density	Ground Yarn Plies	Back-Coating Time	Pile Height (mm)	Stitch Density (Course × Wale) (/cm^2^)
L2-0	Low	2	None	10	9.0 × 6.0
L3-0	Low	3	None	10	9.0 × 6.0
M2-0	Middle	2	None	10	9.0 × 6.0
M3-0	Middle	3	None	10	9.0 × 6.0
H2-0	High	2	None	10	9.0 × 6.0
H3-0	High	3	None	10	9.0 × 6.0
H3-1	High	3	Once	10	9.0 × 6.0
H3-2	High	3	Twice	10	9.0 × 6.0
